# Genomic profiles of the Khazar-Period inhabitants of Phanagoria

**DOI:** 10.18699/vjgb-26-77

**Published:** 2026-09

**Authors:** V.S. Okovantsev, A.D. Manakhov, E.V. Rozhdestvenskih, S.E. Khrapov, M.V. Dobrovolskaya, N.G. Svirkina, S.N. Ostapenko, V.D. Kuznetsov, T.V. Andreeva, E.I. Rogaev

**Affiliations:** Sirius University of Science and Technology, Sirius Federal Territory, Krasnodar region, Russia; Sirius University of Science and Technology, Sirius Federal Territory, Krasnodar region, Russia Vavilov Institute of General Genetics of the Russian Academy of Sciences, Moscow, Russia; Sirius University of Science and Technology, Sirius Federal Territory, Krasnodar region, Russia; Sirius University of Science and Technology, Sirius Federal Territory, Krasnodar region, Russia; Institute of Archaeology of the Russian Academy of Sciences, Moscow, Russia; Sirius University of Science and Technology, Sirius Federal Territory, Krasnodar region, Russia Institute of Archaeology of the Russian Academy of Sciences, Moscow, Russia; Sirius University of Science and Technology, Sirius Federal Territory, Krasnodar region, Russia Institute of Archaeology of the Russian Academy of Sciences, Moscow, Russia The State Museum-Preserve “Phanagoria”, Sennoy village, Krasnodar region, Russia; Sirius University of Science and Technology, Sirius Federal Territory, Krasnodar region, Russia Institute of Archaeology of the Russian Academy of Sciences, Moscow, Russia The State Museum-Preserve “Phanagoria”, Sennoy village, Krasnodar region, Russia; Sirius University of Science and Technology, Sirius Federal Territory, Krasnodar region, Russia Vavilov Institute of General Genetics of the Russian Academy of Sciences, Moscow, Russia Centre of Genetics and Genetic Technologies, Faculty of Biology, Lomonosov Moscow State University, Moscow, Russia; Sirius University of Science and Technology, Sirius Federal Territory, Krasnodar region, Russia UMass Chan Medical School, Department of Psychiatry, Shrewsbury, USA

**Keywords:** ancient DNA, genome, paleoanthropology, Phanagoria, massive parallel sequencing, Khazar Khaganate, древняя ДНК, геном, палеоантропология, Фанагория, параллельное секвенирование, Хазарский каганат

## Abstract

The Phanagoria settlement, located on the Taman Peninsula, is a unique archaeological site. The history of the ancient city spans approximately 15 centuries, making Phanagoria a witness to many historical events, including those related to the early history of the Old Rus’ state. The early medieval period of Phanagoria’s history is of particular interest due to its insufficient study. The Phanagoria Expedition of the Institute of Archaeology of the Russian Academy of Sciences, in collaboration with the Phanagoria Museum-Reserve, studied the city’s cultural strata from the 7th–10th centuries. This period of the urban center’s existence is closely linked to the history of the Khazar Khaganate and culminates in the city’s destruction as a result of an enemy attack. During excavations, skeletal remains bearing signs of violent death were discovered. Anthropological analysis revealed Mongoloid features of the individuals discovered. This paper presents the results of a bioinformatics-based analysis of whole-genome sequences from the remains of three individuals from a Khazar cultural layer. Radiocarbon dating was performed for two individuals. High mitochondrial DNA coverage (43- to 96-fold) confirmed its low level of contamination, and determined the mitochondrial chromosome haplogroup. High genome coverage confirmed the authenticity of the analyzed ancient DNA and allowed the determination of the individuals’ sex and Y-chromosome haplogroup. Population analysis was performed using PCA, ancestral component analysis, using ADMIXTURE, and allele frequency correlation analysis, using the f3 statistic. Whole-genome data analysis of the three samples demonstrated the genetic proximity of the studied individuals to the modern Turkic-speaking population of Central Asia, as well as to early medieval nomadic groups of the Eurasian steppe, Hungarian Avars, and Mongols. Principal component analysis revealed the presence of Asian and European genetic components in the genomes of the studied samples. Our study suggests that the genetic profile of the individuals studied was formed by the mixing of the gene pools of people from the eastern regions of the Eurasian Steppe and populations of Western Eurasia. The Asian components are related to those of medieval groups of Mongols and Avars.

## Abstract (ru)

Городище Фанагория, расположенное на территории Таманского полуострова, представляет собой уникальный объект археологического наследия. История древнего города охватывает около 15 веков, что делает Фанагорию свидетелем многих исторических событий, в том числе имеющих отношение к ранней истории древнерусского государства. Раннесредневековый период истории Фанагории представляет особый интерес в связи с ее недостаточной изученностью. Работами Фанагорийской экспедиции Института археологии РАН в содружестве с Музеем-заповедником «Фанагория» были изучены культурные напластования города VII–X вв. Этот период существования урбанистического центра тесно связан с историей Хазарского каганата и завершается гибелью города в результате вражеского нападения. В ходе раскопок обнаружены скелетные останки людей со следами насильственной смерти. Антропологический анализ выявил монголоидные особенности этих индивидов. В данной работе представлены результаты анализа полногеномных последовательностей из останков трех индивидов из культурного слоя хазарского времени с использованием биоинформатических методов. Для двух индивидов было проведено радиоуглеродное датирование. Благодаря высокому покрытию митохондриальной ДНК (от 43- до 96-кратного), подтвержден низкий уровень контаминации и определена гаплогруппа митохондриальной хромосомы. Установлена аутентичность древней ДНК и определены пол индивидов и гаплогруппа Y-хромосомы. Популяционный анализ проведен методом PCA, анализ предковых компонентов – методом ADMIXTURE и анализ корреляции частот аллелей – методом f3-статистики. Анализ полногеномных данных трех образцов показал генетическую близость исследованных индивидов с современным тюркоязычным населением Центральной Азии, а также с раннесредневековыми группами кочевников Евразийской степи, венгерских авар и монголов. Анализ главных компонент показал присутствие в геномах изученных образцов азиатских и европейских генетических компонентов. Наше исследование позволяет сделать вывод, что генетический профиль изученных индивидов сформировался в результате смешения генофондов выходцев из восточных районов Евразийской степи и населения Западной Евразии. Азиатские компоненты родственны таковым у средневековых групп монголов и авар.

## Introduction

Phanagoria is an ancient city located on the eastern shore of the Kerch Strait (Fig. 1), founded by Greek colonists presumably in the 6th century BCE (Chkhaidze, 2012). In the 5th century CE, along with other cities of the Bosporan Kingdom, Phanagoria became part of the Byzantine Empire, which maintained full control over the city until the mid- 7th century CE (Chkhaidze, 2012). The city’s population in the 5th–6th centuries CE is reported to have been predominantly Greek (Chkhaidze, 2012).

**Fig. 1.**
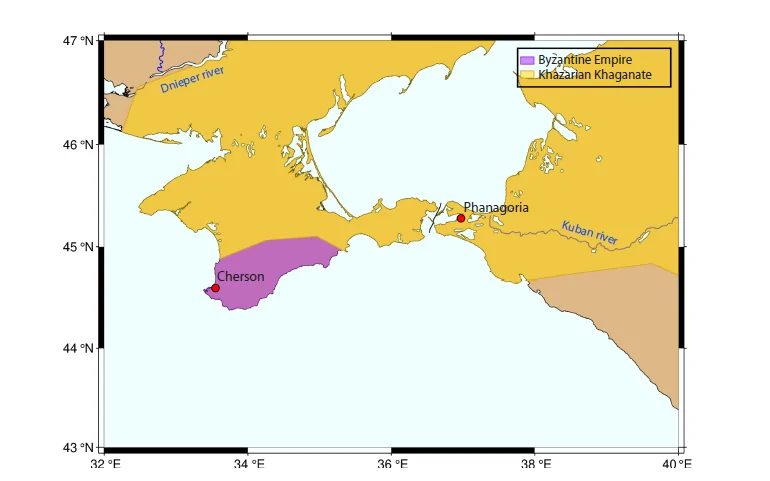
Location of the archaeological site of Phanagoria in the Northern Black Sea region (second half of the 7th century CE). The approximate boundaries of Byzantine and Khazar influence in the region are indicated (according to: Novoseltsev, 1990; Aibabin, 2013).

With the collapse of the Western Turkic Khaganate, the Khazar Khaganate emerged in the Caspian steppes; in the 7th century CE, its influence expanded westward into the Northern Black Sea region and Eastern Azov region (Artamonov, 1962; Golden, 1980). By the end of the 7th century CE, Phanagoria had become an important site of diplomatic contacts between Byzantium and Khazaria. Constantinople retained authority over the city, while two Khazar officials were also active there. This arrangement formed a so-called condominium between the two states. In the 8th century CE, there was a mass migration of the Khazar population to Crimea and the Azov region, caused by clashes between the Khazars and the Arab Caliphate in the Caucasus (Sazanov, Mogarichev, 2006). In the second half of the 9th century CE, Byzantium once again regained control over Phanagoria due to the weakening of Khazaria’s positions under the pressure of the Magyars and Pechenegs. By the beginning of the 10th century CE, the city was devastated by nomads: Magyars, Pechenegs, or Oghuz (Dunlop, 1954; Pletneva, 1982; Novoseltsev, 1990).

The population of the Khaganate was highly diverse and included Bulgars, Alans, Jews, Slavs, and other ethnic and cultural groups. Our knowledge about the Khazars themselves is limited and contradictory (Artamonov, 1962; Balabanova, 2013; Ginzburg, 1963). According to the available linguistic data and information from medieval historians, the Khazars are classified as Turkic-speaking peoples. A relationship between the Khazar language and Bulgar has also been proposed (Novoseltsev, 1990). Among Soviet and Russian authors, there is a widespread hypothesis that the Khazar ethnic group was formed with the participation of the Iranian population of the North Caucasus and incoming Turks, as well as migrants from Siberia (Artamonov, 1962; Novoseltsev, 1990; Balabanova, 2013).

At present, the beginning of the Khazar period in Phanagoria is dated to the last quarter of the 7th century CE (Kuz-netsov, Ostapenko, 2024). The Khazar presence is attested by archaeological finds, including ceramics and architectural remains. In the layers of the 8th–9th centuries CE, ceramics of the Saltovo-Mayaki culture are found (Chkhaidze, 2012), which are associated with the Alanian and Bulgarian components in the population of the Khazar Khaganate (Pletneva, 1967).

The late 8th century CE is particularly important in Khazar history, as this was the period when the Khazar elite adopted Judaism, likely under the influence of a significant Jewish diaspora that existed in the cities of the khaganate (Artamonov, 1962; Kalinina, 2010). It is not possible to determine the proportion of the Jewish population in the Khazar Khaganate. Many researchers believe that only the elite close to the khan were Jewish, while the majority of the population remained pagan (Artamonov, 1962; Novoseltsev, 1990; Kalinina, 2010). Historical sources indicate that there was a Jewish diaspora in Phanagoria during the 1st to 5th centuries CE, as evidenced by numerous Jewish burials (Chkhaidze, 2012). A synagogue was discovered, which existed until its destruction by fire in the 6th century CE (Kuznetsov, 2024). The community also persisted in the Bosporus region during the Khazar period (Chichurov, 1980); however, to date, there is only one archaeological confirmation of this in the form of an amphora with an ancient Hebrew inscription (Golofast, 2020).

According to anthropological data, the population of the Lower Don and Lower Volga regions during the Khazar Khaganate period is characterized by a mixture of Mongoloid and Europid traits (Ginzburg, 1963; Batieva, 2002; Balabanova, 2006, 2013). It is noted that skulls with Mongoloid features are more frequently found in the southernmost sites of the Saltovo-Mayaki culture (Balabanova, 2005, 2006). In burial mounds with ditches, which are associated with the Khazar ethnic group (Semenov, 1983; Novoseltsev, 1990; Afanasyev, 2015), predominantly Mongoloid-type skulls are found (Ginzburg, 1946; Batieva, 2002).

Current studies of the burials in ancient Phanagoria (Manakhov et al., 2025; Rozhdestvenskikh et al., 2026) note the similarity of the population of the Hellenistic and Roman periods with modern European populations. Individuals from ancient Phanagoria have been found to possess mitochondrial DNA and Y-chromosome haplogroups that are common in modern European populations. As a result of the analysis of autosomal markers using the principal component method, individuals from the ancient period are projected to be close to modern European and Caucasian populations

We do not have anthropological materials from the burials of Phanagoria from the Khazar period. All available remains for study come from the cultural layers of the city. The damage identified on the bones indicates an episode of brutal aggression, manifested in the killing of people of different genders and ages. Since the completeness of the materials is far from full, the racial diversity of the sample could only be assessed for a few individuals. Many are characterized by Mongoloid features: a flattened face, slight protrusion of the nasal bones (Dobrovolskaya, Svirkina, 2018).

At present, paleogenetic data on the population of the Khazar Khaganate remain extremely limited. The limited available whole-genome data for Bulgar and Alan samples of the Saltovo-Mayaki culture (14 individuals) have been presented within the framework of studies on the population of the Pontic steppe (Damgaard et al., 2018; Saag et al., 2025).

In this study, we apply genomic and population genetic methods for the first time to remains from Phanagoria dating to the period of Khazar rule in the region.

## Materials and methods

The study included the skeletal remains of four individuals discovered in the “Lower City” excavation of the archaeological site of Phanagoria in 2016, dating back to the period of Khazar Khaganate rule in the Northern Black Sea region during the 7th–10th centuries CE. Anthropological descriptions of the samples and associated archaeological finds are provided in (Dobrovolskaya, Svirkina, 2018). The KhT02 individual is described as an 8–9-year-old girl. Cranial injuries and signs of chronic disease were noted. Cranially, facial flattening is noted. In the KhT03 individual, a chronic inflammatory process has been described, which could have caused developmental delays. The individual was estimated to be 6–7 years old, and sex could not be determined anthropologically. The KhT04 individual is identified as a 45-year-old male, with a healed parietal bone injury and an inflammatory process. Anthropologically, brachycephaly and facial flattening are described. The KhT05 individual was excluded from the analysis due to low endogenous DNA content.

Radiocarbon dating of two studied samples was performed at the Isotope Research Laboratory of the Center for Collective Use “Cenozoic Geochronology” of the Institute of Archaeology and Ethnography, Siberian Branch of the Russian Aca-demy of Sciences, and at the Center for Collective Use “Accelerator Mass Spectrometry” of Novosibirsk State University.

Ancient DNA was extracted from bone fragments at Sirius University in dedicated clean rooms for ancient DNA work, following a previously developed protocol (Andreeva et al., 2022a). Each DNA extraction was accompanied by a negative control. Fragmented genomic libraries were prepared according to a protocol based on the use of single-stranded DNA (Gansauge, Meyer, 2013). DNA extracts and prepared libraries were assessed qualitatively and quantitatively using capillary gel electrophoresis on the Agilent TapeStation 4150 device with the High Sensitivity D1000 reagent kit (Agilent). For the KhT03 sample, a fragment library was additionally prepared from the dDNA preparation, which was subjected to repair using the PreCR Repair Mix (NEB) reagent kit (Mouttham et al., 2015). Sequencing was conducted at the Sirius University on the Illumina MiSeq platform in paired-end read mode of 76+76 nucleotides (test sequencing) and on the Illumina Nova- Seq 6000 platform in single-end read mode of 56 nucleotides (deep sequencing).

Adapter sequences and low-quality reads were removed from the sequencing data using the AdapterRemoval v2 program (Schubert et al., 2016). The resulting reads were then mapped to the human reference genome GRCh37 and the mitochondrial reference genome NC_012920.1 using the BWA program (Li, Durbin, 2009) with parameters adapted for ddDNA sequencing results (Schubert et al., 2012). Using the MarkDuplicates function from the Picard software package (Picard Tools – By Broad Institute, 2019), duplicates were marked to exclude the corresponding reads from further analysis.

Ancient DNA authenticity was assessed using MapDamage2 (Jónsson et al., 2013). Genetic sex was determined using the karyo_RxRy program (Anastasiadou et al., 2024).

The assessment of sample contamination levels was conducted based on mtDNA heterozygosity using the Schmutzi (Renaud et al., 2015) and ContamMix (Fu et al., 2013) programs. For the male sample, an additional analysis of contamination levels based on X chromosome heterozygosity (Rasmussen et al., 2011) was conducted.

The reconstruction of the mitochondrial DNA sequence was carried out by identifying polymorphisms in it using the BCFtools program (Danecek et al., 2021). The identified polymorphisms were filtered by quality (QUAL > 30) and visually checked using the IGV (Integrative Genomics Viewer) program (Thorvaldsdóttir et al., 2013). The determination of mtDNA haplogroups was conducted using the Haplogrep3 program (PhyloTree version 17 – Forensic Update 1.2) (Schönherr et al., 2023), with additional verification of the identified variants using the Yfull MTree 1.02 database (Van Oven, Kayser, 2009).

Phylogenetic analysis of mtDNA sequences was conducted using the mtPhyl software package (Eltsov, Volodko, 2016). For phylogenetic analysis, a sample of mtDNA sequences belonging to the haplogroup identified in the studied individual was formed, using databases containing both ancient and modern samples: HaploTree (HaploTree, 2025), AADR (Mallick et al., 2024), NCBI BLAST (https://blast.ncbi.nlm. nih.gov/Blast.cgi), Yfull MTree 1.02, and AmtDB (Ehler et al., 2019). In addition, databases of ancient samples previously compiled at NTU Sirius (Andreeva et al., 2022b, 2023) were used. Regions of poly-C tracts, tandem repeats, and positions with high mutation frequency were excluded from the analysis.

Using the pileupCaller program from the SequenceTools package (Schiffels, 2025), we reconstructed pseudohaploid genomic profiles of the studied individuals based on 1.24 million genetic markers corresponding to the AADR panel (parameter --randomHaploid). For the analysis, reads with a mapping quality (MQ) and read quality (BQ) of at least 30 were used.

Genetic relatedness was assessed using READv2 (Alaçamlı et al., 2024), which can infer relationships up to the third degree.

Population analysis was conducted using the principal component method (PCA) with the smartpca function from the EIGENSOFT software package (Patterson et al., 2006). The genomic profiles of the individuals studied were projected into the principal component space formed based on the genetic profiles of 2,598 representatives from 168 modern groups from the Eurasian region in the Human Origins array, which includes 600 thousand genetic markers. The analysis was conducted using genomic profiles from which transitions (C ↔ T and G ↔ A), which may be a result of postmortem modifications characteristic of ancient DNA, were excluded.

To compare the studied samples with ancient populations, 1,435 samples from 90 ancient groups of the early Iron Age to medieval periods from Eastern Europe, the Near East, and Central Asia were included in the analysis, as represented in the AADR v.62 database and publications (Nikitin et al., 2025; Saag et al., 2025).

The ADMIXTURE analysis was conducted using a set of 560 thousand autosomal genetic markers from the AADR v.62 database and materials from publications (Lazaridis et al., 2014; Flegontov et al., 2019; Nikitin et al., 2025; Saag et al., 2025). The ADMIXTURE v1.3 program (Alexander et al., 2009) was used with default parameters. We conducted 10 independent calculations, varying the number of assumed ancestral components (K) from 1 to 12. The optimal number of components was determined based on the comparison of CV-error values calculated by the program; as a result, the optimal value was K = 5. Out of 10 options, the calculation with the minimum CV-error value was selected (Supplementary Materials)1.

Supplementary Materials are available in the online version of the paper: https://vavilovj-icg.ru/download/pict-2026-30/appx42.xlsx


Before the analysis, sample selection and genetic variant filtering were conducted. In cases where the samples originated from the same family group, only one individual with the highest number of identified autosomal pseudodiploid genotypes was included in the analysis. Using the PLINK v2.00 program (Chang et al., 2015), genetic variants were excluded from the analysis if their genotypes were determined in only one individual (--geno 0.999); variants with a minor allele frequency of less than 0.002 (--maf 0.002); variants that are transitions, which may be a result of post-mortem modifications characteristic of ancient DNA; as well as linked genetic variants (--indep-pairwise 200 5 0.5). Samples for which fewer than 10 thousand pseudodiploid markers remained after filtering were excluded from the analysis.

A total of 2,521 samples were included in the analysis, including three representatives of the population of Phanagoria from the Khazar period; 2,248 ancient samples from the AADR v62 database and published materials (Flegontov et al., 2019; Lazaridis et al., 2025; Nikitin et al., 2025; Saag et al., 2025) from the period ranging from the Early Bronze Age to the Middle Ages in Eurasia; 188 ancient and 85 modern samples from the AADR v62 database, representing groups in which commonly accepted ancestral components in paleogenetic studies are maximized (WHG (Western European Hunters Gatherers) – component associated with Western European Mesolithic hunter-gatherers; EHG (Eastern European Hunters Gatherers) – component associated with Eastern European Mesolithic hunter-gatherers; ANF (Anatolian Neolithic Farmers) – component associated with Neolithic farmers of Anatolia; Han – East Asian component maximized in the modern Han Chinese population; Nganasan – Siberian component maximized in the modern Nganasan population; Mbuti – African component represented by the African Mbuti population) (see further).

To assess genetic similarity with previously published ancient populations, f3-statistics were calculated. Pseudohaploid genotypes from a panel of 1.24 million genetic markers were included in the analysis, with transitions being excluded. The f3-statistic calculations were performed using the Admix- Tools v.7.0.1 (Patterson et al., 2012) and admixr v.0.9.1 (Petr et al., 2019) software in the form f3 (Mbuti, Test; KhT02/ KhT03/KhT04), where Test is one of the ancient groups relative to which the sample under study is analyzed; Mbuti are representatives of the African Mbuti population used as an external group. Ancient groups from Europe, Central, and East Asia dating on average from 1,700 to 700 years ago were taken as the test. Only groups represented by more than three non-related individuals and having at least 20 thousand genetic markers overlapping with the studied samples KhT02/KhT03/ KhT04 were included in the analysis.

## Results

Radiocarbon dating of two samples confirmed that they date to the Khazar period (Table 1). The two obtained radiocarbon dates have very broad calibrated ranges. Both KhT02 and KhT03 are associated with the same episode of violence; however, the radiocarbon age and calibrated dates show a significant temporal discrepancy between these samples. This discrepancy calls for a cautious interpretation of the dates and for expanding both the number of dated samples and the range of dated materials (Table 1). Nevertheless, the attack on the city is more likely to date to the 8th century CE than to the 9th century CE; therefore, linking these deaths to the final destruction of the city is currently unlikely.

**Table 1.**
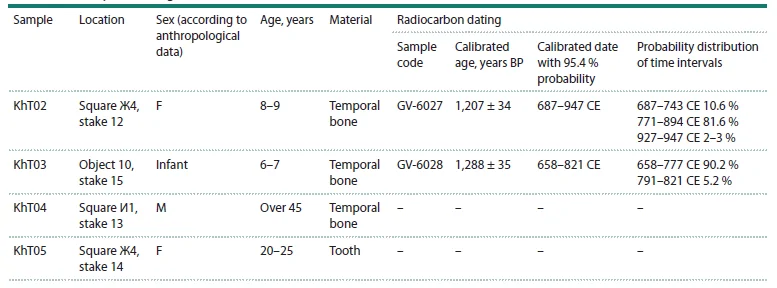
Archaeological and anthropological data on the examined samples from the 2016 excavation of the “Lower City” of Phanagoria

Genomic DNA was successfully extracted from bone samples of all four individuals and used to prepare genomic libraries for sequencing. In three samples, a high proportion of reads mapped to the human reference genome and aDNA authenticity were confirmed, allowing the use of the DNA from these samples for whole-genome analysis (Table 2, Fig. 2). Sample KhT05 was excluded from further analysis due to low endogenous DNA content.

**Table 2.**
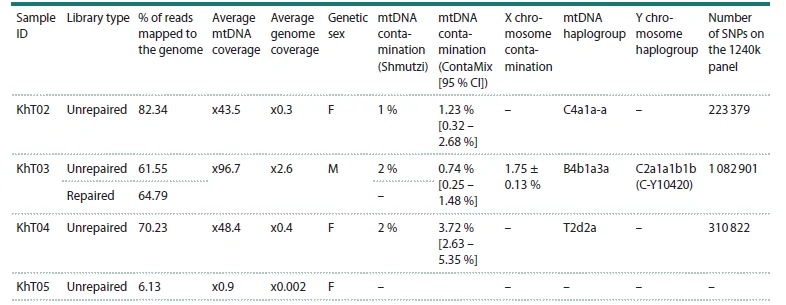
Statistics of DNA sequencing, mtDNA and Y-chromosome haplogroups

**Fig. 2.**
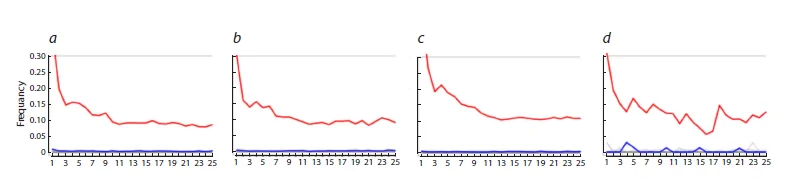
Profiles of nucleotide substitutions for reads mapped to the reference mtDNA sequence, calculated using MapDamage2. C > T substitutions specific to mtDNA are shown with a red line. The X-axis marks the nucleotide position from the 5’ end of the DNA fragments. Samples: a – KhT02; b – KhT03; c – KhT04; d – KhT05.

For all three samples, the genetic sex was determined: samples KhT02 (child aged 8–9 years) and KhT04 (individual over 45 years old) were female, while sample KhT03 (child aged 6–7 years) was male. The discrepancy between the anthropological and genetic sex estimates for KhT04 is noteworthy. Based on the preserved morphological features of the skull (development of the supraorbital ridge, outer edge of the eye socket, size of the mastoid process, overall robustness of the skull), as well as the size and prominence of the relief on the bones of the upper limb, the skeleton was determined to belong to a male. It should be noted that anthropological traits provide a probabilistic determination of biological sex, so discrepancies in the results of sex diagnosis by two methods are possible. In this case, the robust physical habitus of an individual genetically identified as female is noteworthy.

The assessment of contamination levels for the male sample based on mtDNA and the X chromosome showed that the contamination does not exceed the permissible 5 % level for paleogenomic studies (Table 2)

For all samples, complete mtDNA sequences were reconstructed and their mitochondrial haplogroups were determined (KhT02 – C4a1a-a, KhT03 – B4b1a3a, KhT04 – T2d2a) (Fig. 3). Since the haplogroups differ, the studied individuals are unlikely to have been direct maternal relatives.

**Fig. 3.**
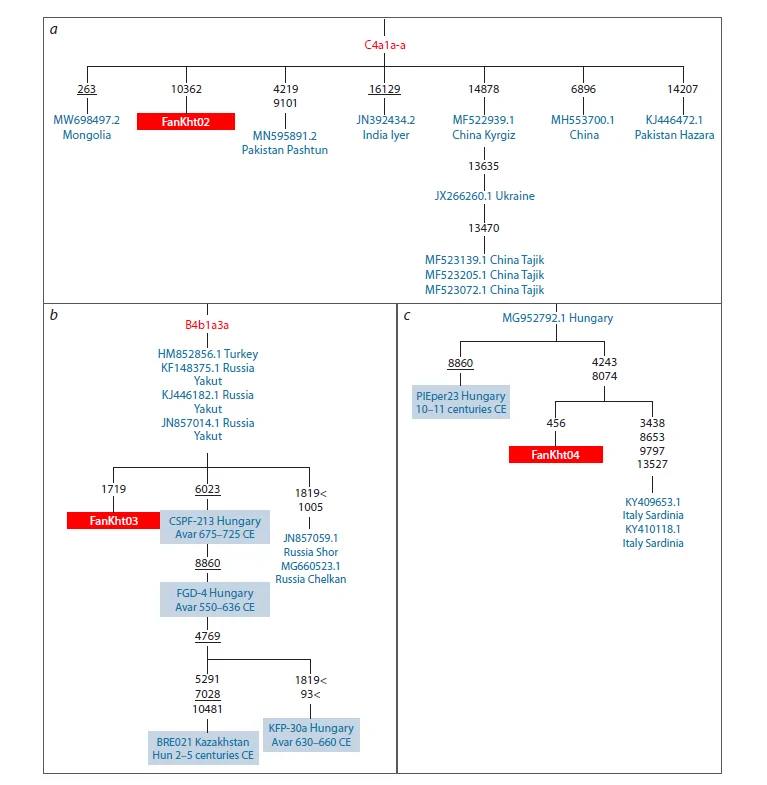
Phylogenetic tree of haplogroups: a – C4a1a-a; b – B4b1a3a; c – T2d2a, constructed using the mtPhyl software package. Mutations on the branches are indicated relative to the reference mtDNA sequence (rCRS). For transitions, only the nucleotide position number is indicated, while for transversions, the nucleotide replacement is provided; positions of recurrent mutations are marked with the symbol “<”. Uncertain positions in the mtDNA sequences were not considered when constructing the tree. Ancient samples are highlighted with a blue background. Geographic origin is indicated for each sample, and chronological age is provided for ancient samples (Table 2).

The kinship analysis based on whole-genome sequencing data, conducted using the READv2 program, also showed that the studied individuals are not first, second, or third-degree relatives

For the male individual KhT03, the Y-chromosome haplogroup was determined as C-Y10420 (C2a1a1b1b).

Population analysis conducted using the principal component method showed that the studied samples project near the modern Turkic-speaking populations of Central Asia – Turkmen, Uzbeks, Karakalpaks, and Kazakhs – as well as the Nogais of the southern European part of Russia (Fig. 4a). At the same time, all three samples are separated from each other and are located along the Caucasus–Mongolia axis.

**Fig. 4.**
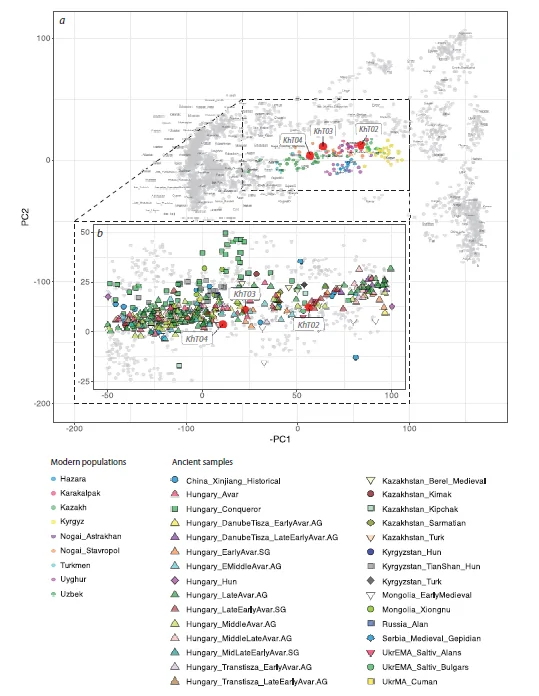
Projection of genomic profiles of the studied Khazar-period individuals from Phanagoria (KhT02–KhT04) (marked in red) in the principal component space (PC1 and PC2), formed based on the genetic profiles of representatives of modern Eurasian populations (gray points), with colored points marking samples of modern Hazaras, Karakalpaks, Kazakhs, Nogais, Turkmen, Uyghurs, and Uzbeks (a); projection of genomic profiles of the studied individuals with ancient groups from open sources with an average dating from 1,700 years ago to 700 years ago (b).

Sample KhT04 is located closer than the others to the cluster of European groups, in the array of Turkmen, Uzbeks, and Stavropol Nogais. Sample KhT02 clusters with Kazakhs, Kyrgyz, and Karakalpaks. KhT03 is projected between them alongside Astrakhan and Stavropol Nogais. It can be noted that all three studied samples do not intersect with the cluster of modern Jewish populations, which group with other populations of the Middle East and Levant.

When compared to ancient populations of the early Iron Age, early and high Middle Ages (Fig. 4b), the studied samples cluster with early Iron Age and medieval steppe nomads from the territories of present-day Kazakhstan, Mongolia, China, and Hungary. All individuals project onto the Avar cluster from the territory of Hungary.

To determine the ancestral components constituting the genomes of the studied samples from Phanagoria, an ADMIXTURE analysis was conducted in the context of ancient Neolithic, Bronze Age, Iron Age, and medieval populations (Fig. 5). In the KhT samples, four components are identified, named according to the groups in which each predominates: Anatolian Neolithic Farmers (ANF) (Turkey_ Marmara_Barcin_N.AG), European Hunter-Gatherers (Europe HG) (France_Mesolithic.AG, Russia_YuzhniyOleniy Ostrov_Mesolithic.AG), East Asian (Han), and Ancient Siberian component (Nganasan).

**Fig. 5.**
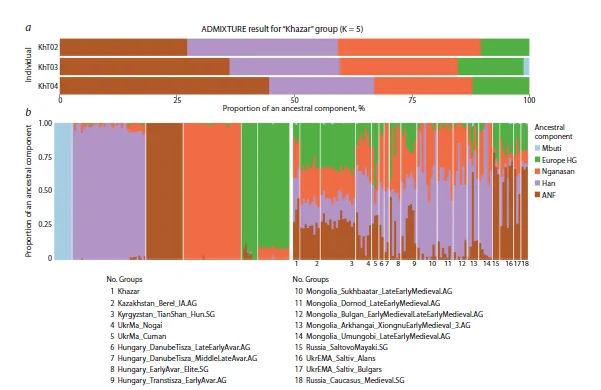
ADMIXTURE results (K = 5). a – for the studied samples KhT02–04; b – for six groups, where one of the ancestral components is maximized, and for some groups of Iron Age and medieval nomads (Supplementary Material 1).

Analysis using the f3-statistics method showed that samples KhT02 and KhT03 have genetic profiles similar to those of Avars from Hungary (Table 3, Fig. 6). For the KhT03 individual, a similarity is observed with the group Avar_Asia_Core2, representing early Avars from the 6th–8th centuries from the cemeteries of Apátfalva-Nagy-út-dűlő, Budapest-Csepel- Kavicsbánya, Csólyospálos-Felsőpálos, Fajsz-Garadomb, Felgyő-Ürmöstanya, Kiskőrös-Vágóhídidűlő, Kunpeszér- Felsőpeszériút-Homokbánya, Makó-Mikócsa-halom in Hungary with a predominant East Asian component (Maróti et al., 2022). For the KhT04 individual, a high value of f3-statistics, in addition to Avars, is observed for the group of the medieval population of South Mongolia from the cemeteries of Banzart- Khairkhan, Erdene-Mountain, Ganzagad, Gun-Tarmagtai, Ikh-Uvgun, Shar-Tolgoi in Umungobi Province, Mongolia (Mongolia_Umungobi_EarlyMedieval), as well as for the Cumans from the cemeteries of Mamai-Gora and Velika Znamyanka in Zaporizhzhia and Kumy in Kharkiv Oblast (UkrMA_Cuman) (Saag et al., 2025).

**Table 3.**
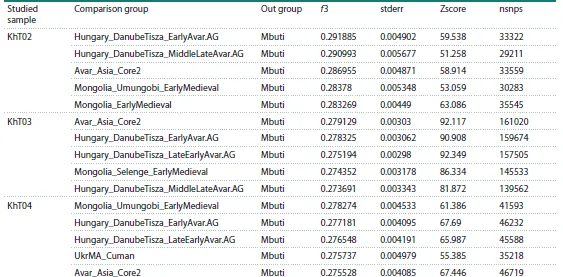
Values of the f3-statistic f3(KhT, Test; Mbuti.DG) for Khazar period samples and comparison groups Notе. stderr (standard error) is the standard error of the f3-statistic estimate, characterizing the precision of its calculation; Z-score is the ratio of the f3 value to its standard error (f3/stderr), reflecting the statistical reliability of the estimate — the higher the value, the more reliable the result; nsnps is the number of single-nucleotide polymorphisms (SNPs) used to calculate the f3-statistic for a given pair of samples

**Fig. 6.**
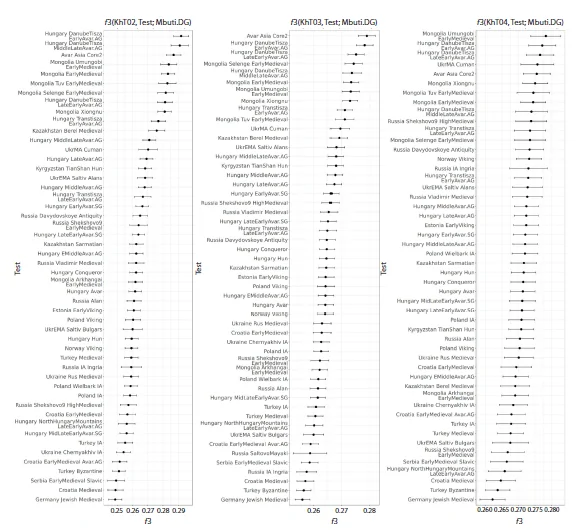
f3-statistics for samples KhT02, KhT03, and KhT04 compared with ancient groups from the Early Iron Age to the Middle Ages. The higher the f3 values, the more genetically similar the comparison group and the studied sample are.

## Discussion

Studies of skeletal remains from small burial mounds near the Khazar city of Sarkel, in the area of the modern Tsimlyansk Reservoir, reported southern Siberian traits characteristic of Turkic and Cuman groups (Ginzburg, 1946). In the burial mounds of the Lower Don region, associated with the ethnic Khazars (Semenov, 1983; Novoseltsev, 1990), the buried individuals were predominantly described in the anthropological literature as having Mongoloid cranial features, and “male Mongoloid skulls and skulls from female samples show the greatest similarity to the Huns of Transbaikalia and the nomadic Turks of Siberia, Altai, and Kazakhstan” (Batieva, 2002, p. 99). Anthropological studies of remains from Phanagoria during the Khazar period note the presence of a Mongoloid component (Dobrovolskaya, Svirkina, 2018).

PCA results for the studied Khazar-period samples from Phanagoria, in the context of modern and ancient populations, confirm the conclusions of anthropology and support the presence of an Asian-related component in their genomes. The genetic profile of samples from the Khazar period in the Lower City of Phanagoria is similar to that of modern Turkicspeaking peoples of Central Asia and the historical nomadic population of the Eurasian steppe, but this similarity does not necessarily indicate direct common origin.

The steppe population was formed as a result of complex processes involving carriers of Asian and European genetic components. The position of the KhT samples in PCA space likely reflects admixture processes similar to those that shaped the genomes of steppe populations. At the same time, it should be noted that the three studied samples are located at a certain distance from each other in the principal component space. Sample KhT04 is closer than the others to the cluster of groups with a known presence of West Eurasian roots, KhT02, to the steppe Turkic groups (Kazakhs, Kyrgyz), and KhT03 is in between them. Comparison with ancient samples showed that all three samples fall into a large cluster of Avars from the territory of Hungary (550–900 CE).

Analysis of mtDNA uniparental markers (Fig. 3) showed that the KhT02 sample belongs to haplogroup C4a1a-a (which diverged about 3.4 thousand years ago in Southern Siberia (Derenko et al., 2010; YFull, 2025)), which is widespread among the modern populations of China, Siberia, Afghanistan, and Pakistan (Van Oven, Kayser, 2009; YFull, 2025), and was also found in the population of the Khovsgol Mongolian aimag of the Bronze Age, among the Huns and Xiongnu of the early Iron Age from the territory of Kazakhstan, early medieval Visigoths from the territory of Spain, and the population of the Irkutsk region of the early Bronze Age. Phylogenetic analysis showed that the sequences most closely related to the mtDNA of this individual are found in modern samples

Sample KhT03 belongs to haplogroup B4b1a3a (which diverged about 6.8 thousand years ago (YFull, 2025)), which is widespread among the modern populations of Southern and Eastern Siberia, while among ancient groups it was found in the Avars of the 6th–9th centuries from Hungary, in the early medieval Huns from the Berel burial ground in Kazakhstan, and in the early Iron Age population of Mongolia. The mtDNA sequences most closely related to the mtDNA of the KhT03 individual were found in modern populations of Siberia, Turkey, as well as among the Avars.

Sample KhT04 belongs to haplogroup T2d2a (which diverged about 5.4 thousand years ago (YFull, 2025)), which is widespread in modern Turkey, the Mediterranean, and the Caucasus. Among ancient groups, this lineage was found in individuals from late antique Rome, from the Iranian burial sites of Dinkha-Tepe and Shahr-i Sokhta of the Bronze Age and the Iron Age burial site of Hasanlu, an individual from the Early Bronze Age Lake culture in Bulgaria, an Etruscan from the Early Iron Age in Italy, and a Magyar from the 10th–11th centuries in Hungary. Phylogeographic analysis showed that the mtDNA sequence of the KhT04 sample is most closely related to modern samples from Hungary and Italy, as well as to a medieval sample (10th–11th centuries) from Hungary.Thus, both based on the analysis of mtDNA uniparental markers and the analysis of autosomal profiles of the studied samples, KhT02 and KhT03 show affinities with Central Asian and Siberian populations, whereas KhT04 shows stronger affinity with West Eurasian populations, whose descendants migrated to Europe as a result of migration processes in the early Iron Age.

ADMIXTURE analysis indicates that more than half of the ancestry of the studied Phanagoria samples is represented by Asian-related components: the East Asian Han component and the ancient Siberian Nganasan, which are represented in equal proportions in the genomes of medieval samples from Mongolia (Mongolia_Sukhbaatar_LateEarlyMedieval.AG, Mongolia_Dornod_LateEarlyMedieval.AG). Moreover, significant proportions of the components Europe HG and ANF, which are widespread in European and Near Eastern groups, as well as found among the nomadic groups of Central Asia, are also present.

The proportions of these components make the Phanagoria samples most similar to Avar groups of the 6th–9th centuries from the cemeteries in northeastern Hungary, Hungary_ DanubeTisza_LateEarlyAvar.AG and Hungary_Transtisza_ EarlyAvar.AG, which may indicate similar admixture processes. The difference in the proportions of the ANF component between the studied individuals corresponds to their positions on the PCA projection: the individual KhT04, clustering closer to the European clusters, has a higher proportion of the Anatolian ANF component, whereas the individual KhT02 has a lower proportion of this component, which aligns with its position on the PCA graph. The increase in the proportion of ANF may be the result of mixing with groups from the Black Sea and Transcaucasia, where this component predominates, as seen in the studied groups of Alans from the Saltovo-Maiaki culture (Russia_SaltovoMayaki.SG) and the medieval population of Anapa (Russia_Caucasus_Medieval.SG).

The f3 results show that all three samples have genetic affinities with early and elite Avar groups, which exhibit stronger Siberian and East Asian components than others. This may suggest a shared East Asian-related ancestry component for the Avars and the ancestors of the studied samples. The presence of similarity with the UkrMA_Cuman group, which has a component profile characteristic of nomadic groups in the western part of the Eurasian steppe (Saag et al., 2025), in individual KhT04 indicates this individual’s mixed ancestry involving Asian and European-related components.

The small number of synchronous samples does not allow us to definitively assert whether the genetic profile of individuals KhT02–04 is typical for representatives of the Khazar community itself or another Turkic group of the early Middle Ages living in the territory of the khaganate. These individuals differ sharply from previously studied individuals from ancient Phanagoria (Manakhov et al., 2025; Rozhdestvenskikh et al., 2026), who show similarities with European populations. These genetic differences are consistent with the cultural and demographic changes documented in the history of the city.

In the Northern Black Sea region, Khazaria emerged after the collapse of the Western part of the Turkic Khaganate, whose population included genetically diverse components. Therefore, the study of the genetic structure of Khazaria is an extremely complex task. Previous cranial studies also emphasized this complexity, identifying several distinct anthropological components (Ginzburg, 1963). Moreover, cranial features demonstrate a connection with burial rites, indicating a relatively isolated existence of certain groups within Khazar society. Our materials do not allow us to determine whether the individuals included in the genetic analysis belonged to different social, ethnic, or cultural groups. Therefore, at the current stage of research, we find it difficult to link the genetic heterogeneity of the studied individuals with their original heterogeneity or with the existing differences between groups within Phanagoria during the Khazar period.

## Conclusion

Our study supports previous anthropological observations indicating the presence of an Asian-related component among the inhabitants of Phanagoria during the Khazar period. The admixture processes underlying this genetic profile may have been similar to those observed in the Avars, who carried an East Asian-related substrate combined with West Eurasian components. However, in the absence of available wholegenome data from securely identified ethnic Khazars, it is not yet possible to determine whether the studied individuals belonged to the Khazars themselves or to other early medieval nomadic groups.

## Conflict of interest

The authors declare no conflict of interest.
